# Diagnostic challenges between takotsubo cardiomyopathy and acute myocardial infarction—where is the emergency?: a literature review

**DOI:** 10.1186/s12245-024-00595-4

**Published:** 2024-02-15

**Authors:** Alexandru Scafa-Udriste, Ruxandra-Nicoleta Horodinschi, Miruna Babos, Bogdan Dinu

**Affiliations:** 1 “Carol Davila” University of Medicine and Pharmacy, Bucharest, 050474 Romania; 2 Department of Cardiology, Clinical Emergency Hospital of Buchararest, Bucharest, 014461 Romania; 3 Department of Emergency Medicine, Clinical Emergency Hospital of Buchararest, Bucharest, 014461 Romania

**Keywords:** Takotsubo cardiomyopathy, Acute myocardial infarction, Echocardiography, Therapeutic strategy, Prognosis

## Abstract

**Background:**

Takotsubo cardiomyopathy (TC) is an emergency cardiovascular disease, with clinical and paraclinical manifestations similar to acute myocardial infarction (AMI), but it is characterized by reversible systolic dysfunction of the left ventricle (LV) in the absence (most of the time) of obstructive coronary artery disease (CAD).

**Management of patients with TC:**

TC seems to be more frequent in post-menopausal women and it is triggered by emotional or physical stress. The diagnosis of TC is based on the Mayo Clinic criteria. Initially, patients with TC should be treated as those with AMI and carefully monitored in intensive care unit. Urgent clinical and paraclinical distinction between TC and AMI is mandatory in all patients, because of the different therapeutical management between the two diseases. Chest pain and dyspnea are the most common symptoms in TC. Paraclinical diagnosis is based on cardiac biomarkers, electrocardiogram (ST-segment elevation/T wave inversion in precordial leads without reciprocal ST-segment depression in inferior leads and absence of Q waves), echocardiography (LV systolic dysfunction, regional wall motion abnormalities extended in more than one coronary territory), cardiac magnetic resonance and in most of the cases the positive diagnosis is established by performing CA to exclude obstructive CAD. The prognosis of patients with TC is considered benign in most cases, with a complete LV function recovery, but severe complications may occur, such as cardiogenic shock, LV free wall rupture, life-threatening arrhythmia, and cardiac arrest. Postoperative TC may develop after any type of surgical intervention due to acute stress and it should be differentiated from postoperative AMI. The management of patients with TC is medical and it is based on supportive care and the treatment of heart failure, while patients with AMI require myocardial revascularization.

**Conclusions:**

TC leads to transient LV dysfunction that mimics AMI from which it should be differentiated for a good therapeutic approach. Patients with TC should be carefully monitored during hospitalization because they have a high recovery potential if optimally treated.

## Background

Takotsubo cardiomyopathy (TC) is an acute cardiovascular disease, characterized by reversible systolic dysfunction of the left ventricle (LV) in the absence of obstructive coronary artery disease (CAD). TC is more frequent in post-menopausal women triggered by emotional or physical stress—it is also called stress-induced cardiomyopathy [1]. TC may be underdiagnosed or misdiagnosed being confused with acute myocardial infarction (AMI) at the first presentation of a patient in the emergency room, because of the similarities between the two diseases.

This literature review describes the clinical and paraclinical diagnostic criteria for TC, the treatment options, the prognosis, and the differences between TC and AMI. For this reason, an accurate diagnosis and optimal therapeutic strategy are necessary.

### Epidemiology of TC

The prevalence of TC is about 2–3% of patients presenting with positive troponin suspected of CAD and about 5–6% of women with suspected ST-segment elevation myocardial infarction undergoing urgent coronary angiography (CA) have TC [2]. The prevalence of TC may be even greater than that, because it may be underdiagnosed, especially in patients who associate CAD. It was first described in a group of post-menopausal women in Japan more than 30 years ago, but nowadays the disease is more frequently diagnosed due to the higher possibility of performing CA than before. More than this, during the coronavirus disease-19 pandemic it was reported an increase in the incidence of TC compared to the time before the pandemic [3].

TC is more frequent in women than in men and the majority of patients developing TC are older than 50 years [4]. Patients with TC younger than 50 years old represent about 10% of all patients with TC [2].

Furthermore, women with TC are usually older than 55 years of age and old women have a 5 times higher probability of developing TC compared to young women [5–7]. Young patients are more frequently men with psychiatric or neurological affections and are predisposed to develop complications more frequently [2].

### Diagnosis of TC

Currently, the diagnosis criteria for TC, as proposed by Mayo Clinic, have four components:

- 1. Temporary, reversible hypokinesis, akinesis, or dyskinesis in LV segments with or without apical involvement with a regional wall motion abnormality (RWMA), that exceeds a single coronary artery territory; the presence of an episode of emotional or physical stress;
- 2. The lack of significant of CAD;
- 3. Recent repolarization changes identified on the electrocardiogram (ECG) such as ST-segment elevation and/or T wave inversion or significant increase in cardiac troponin serum level;
- 4. The absence of myocarditis and pheochromocytoma [1].

### Subtypes of TC

**Fig. 1:**
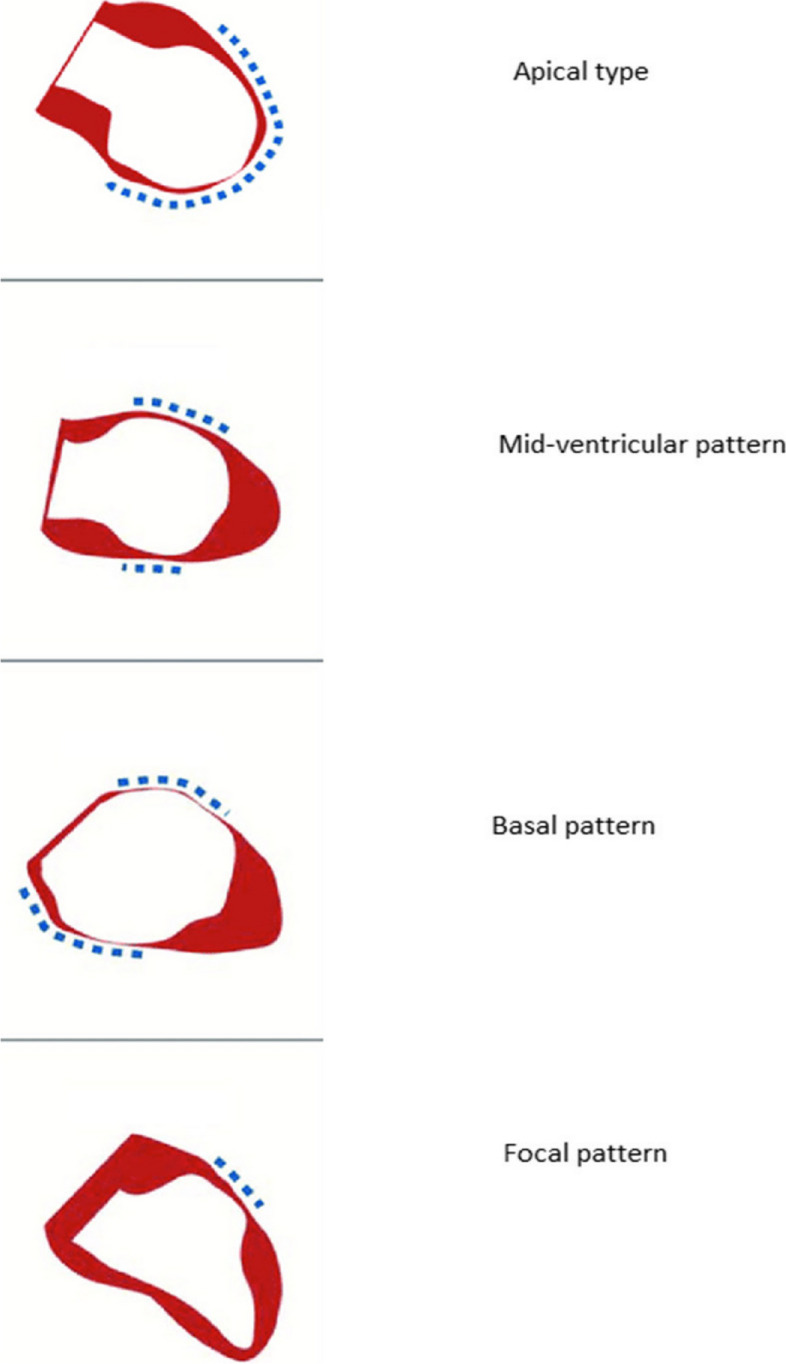
Subtypes of TC

There are described four subtypes of TC depending on the distribution of LV wall motion abnormalities (Fig. 1). The most common subtype is the apical one according to the InterTASK registry [8].

The subtypes of TC are the following:

- Apical ballooning type (81.7%)
- Mid-ventricular wall motion pattern (14.6%)
- Basal wall motion pattern (2.2%)
- Focal wall motion pattern (1.5%) [8].

In Table 1, there are reviewed the differences regarding clinical presentation and risk factors between TC and AMI.

**Table 1:** Differences in clinical presentation and risk factors between TC and AMI

	TC	AMI
1.Clinical presentation		
Symptoms	Chest pain, dyspnea (acute heart failure, pulmonary edema)—most common symptoms Syncope Palpitations: arrhythmias (ventricular fibrillation—uncommon) Asymptomatic	Chest pain Dyspnea (acute heart failure, pulmonary edema) Syncope Palpitations: arrhythmias (ventricular arrhythmias: monomorphic/polymorphic ventricular tachycardia, ventricular fibrillation)
Signs Systolic murmur	Mitral regurgitation Dynamic LVOTO/LV intraventricular obstruction LV free wall rupture	Mitral regurgitation LV free wall rupture
Pulmonary crackles	Acute heart failure/edema pulmonary	Acute heart failure/Oedema pulmonary
Hypotension/hemodynamic instability	Cardiogenic shock—uncommon Life-threatening arrhythmias Cardiac arrest—rare	Cardiogenic shock Life-threatening arrhythmias Cardiac arrest
2.Risk factors		
Cardiovascular risk factors	Usually absent	Smoking, HT, dyslipidemia, diabetes mellitus, obesity
Sex	Women> men	Men> women
Age	elderly, post-menopausal women	Elderly

*AMI* Acute myocardial infarction, *HT* Arterial hypertension, *LV* Left ventricle, *LVOTO* Left ventricle outflow tract obstruction, *TC* Takotsubo cardiomyopathy

## Clinical presentation

Clinical presentation of patients with TC is similar to those with AMI in most cases, this is why the differential diagnosis between TC and CAD is mandatory. The majority of patients present similar symptoms with myocardial ischemia in the acute phase. Chest pain and dyspnea are the most frequent symptoms, but patients may also present with heart failure, pulmonary edema, cardiogenic shock, syncope, life-threatening arrhythmias, such as sustained monomorphic/polymorphic tachycardia, ventricular fibrillation, or even cardiac arrest. Some patients may be asymptomatic, especially those hospitalized for another acute disease, such as sepsis, or surgical interventions. In these cases, the diagnosis is established using the troponin, electrocardiogram (ECG), and echocardiographic abnormalities [2].

The physical examination may be normal, but in some patients may appear symptoms and signs of acute heart failure, pulmonary edema, cardiogenic shock, and arrhythmias. In some cases, we may observe mitral regurgitation murmur due to systolic anterior motion, papillary muscle dysfunction because of LV wall motion abnormalities, and mitral leaflet tethering [2]. Another clinical sign that may appear is the systolic murmur caused by the dynamic LV obstruction tract in the apical pattern TC associated with basal LV wall segment hyperkinesia [2]. The murmur caused by LV free wall rupture may be detected in rare cases.

Taking into account that the clinical examination is unspecific and very similar in patients with TC and in those with TC, a set of paraclinical tests is necessary to differentiate the two diseases.

### Risk factors

Patients with TC are old people, with a mean age of 58–75 years old, especially post-menopausal women, without cardiovascular risk factors [9]. Contrary, patients with AMI are more common men, with multiple typical cardiovascular risk factors, for example, smoking history, arterial hypertension, dyslipidemia, obesity, and diabetes mellitus.

### Paraclinical tests

The paraclinical tests useful to establish the diagnosis of TC are cardiac biomarkers, ECG, transthoracic echocardiography (TTE), CA to exclude obstructive CAD, cardiac magnetic resonance to differentiate from myocarditis or cardiomyopathies, coronary computed tomography angiography in patients with low probability of AMI. The differences regarding paraclinical tests between TC and AMI are described in Table 2.

**Table 2:** Paraclinical tests differences between TC and AMI

	TC	AMI
1.Laboratory tests		
Cardiac biomarkers		
Troponin T/I	Mildly/moderately increased	Markedly increased
BNP, NT-proBNP	Markedly increased	Mildly increased
Inflammatory markers		
Leukocytosis	Present	Present
CRP	increased	Increased
Serum catecholamines	increased	Normal
2.ECG		
ST-segment	-ST-segment elevation > 1 mm in precordial leads without reciprocal ST-segment depression in inferior leads -ST-segment depression—less frequent	-ST-segment elevation in at least 2 contiguous leads with reciprocal ST-depression in inferior leads -ST-segment depression (horizontal/down-slope) > 0.5 mm in 2 contiguous leads
T wave	T wave inversion in the anterior leads	T wave inversion in 2 contiguous leads with R > S
Q wave	Without Q waves	Present
QT segment	Prolonged	Normal
Arrhythmias	Monomorphic/polymorphic ventricular tachycardia Ventricular fibrillation Torsade’s de points	Monomorphic/polymorphic ventricular tachycardia Ventricular fibrillation Torsade’s de points
Atrioventricular block	May be present	May be present
Bundle branch block	Absent	Left/right bundle branch block may be present
1.Echocardiography		
LV systolic function	Acute phase: Temporary reduced Long-term: complete recovery	Acute phase: Reduced Long-term: complete/partial recovery or persistent LV dysfunction
LV wall motion abnormalities	RWMA *not* limited to an epicardial coronary artery territory LV apical ballooning pattern Mid-ventricular pattern ballooning ± SAM—uncommon Basal ballooning pattern—uncommon Focal pattern	RWMA limited to an epicardial coronary artery territory
Complications	LV thrombus Mitral regurgitation LV free wall rupture	Apical aneurysm LV thrombus Mitral regurgitation LV free wall rupture
6. CMR		
*T2*	Acute phase: transmural myocardial edema Subacute phase: fine remaining RWMA	Myocardial edema RWMA
*LGE*	Acute phase: transmural areas of LGE uptake at the hinge between akinetic/dyskinetic ballooning segments and hypercontractile segment, but no persistent LGE on long-term	Focal subendocardial/transmural LGE uptake
EGE	LV thrombi	LV thrombi
7. Coronary computed tomography angiography		
	Normal epicardial coronary arteries/non-obstructive CAD	Obstructive CAD
8. Coronary angiography		
Coronary angiography	Normal epicardial coronary arteries Non-obstructive CAD (stenosis < 50%)	Obstructive CAD (stenosis ≥ 50%) MINOCA
Ventriculography	LV mid- and apical segments akinesis and hypercontractility of the basal segments	–
Invasive hemodynamics	LVOTO (20% of patients) Increased LVEDP	Increased LVEDP

*AMI* Acute myocardial infarction, *BNP* Brain natriuretic peptide, *CAD* Coronary artery disease, *CMR* Cardiac magnetic resonance, *CRP* C-reactive protein, *EGE* Early gadolinium enhancement, *LGE* Late gadolinium enhancement, *LVEDP* Left ventricle end-diastolic pressure, *LVEF* Left ventricle ejection fraction, *MINOCA* Myocardial infarction with non-obstructive coronary arteries, *NT-proBNP* N-terminal pro-BNP, *RWMA* Regional wall motion abnormality, *SAM* Systolic anterior motion, *TC* Takotsubo cardiomyopathy

### Laboratory tests

#### Cardiac biomarkers

Cardiac biomarkers are increased in TC, because of the myocardial dysfunction. Cardiac biomarkers of myocardial necrosis, troponin T, troponin I, and creatinine kinase are elevated. Troponin level is mildly increased in approximately 90% of the patients with TC, leading to a misdiagnosis of AMI, but in TC the increase of the troponin is lower (usually less than 1 ng/mL) compared to patients with AMI [10]. The mild troponin increase is disproportionated to the remarkable ECG changes, LV systolic dysfunction, and important wall motion abnormalities [2].

Natriuretic peptides, B-type natriuretic peptide (BNP), and N-terminal pro-BNP (NT-proBNP) are 3- to fourfold higher in patients with TC than in those with AMI [11]. NT-proBNP is higher in patients with apical TC compared to the other subtypes due to a more important degree of acute LV dilation and myocardial stretch [2]. The elevation of cardiac biomarkers is secondary to LV systolic dysfunction, myocardial stretch, and increased plasma catecholamine levels [11].

Inflammatory markers, such as leukocytosis, increased C-reactive protein are present in both conditions, but C-reactive protein is more increased in TC than in AMI [2]. Proinflammatory cytokines, such as interleukin-2, interleukin-4, interleukin-8, interleukin-10, and tumor necrosis factor-alfa are increased in patients with TC in the acute phase and remain elevated for several months [2]. Contrary interleukin-6 is higher in patients with AMI than in those with TC, because of the greater area of myocardial necrosis [2]. In patients with TC, increased serum levels of catecholamines are noticed.

### Electrocardiogram

Electrocardiogram should be performed in all patients with chest pain, especially to prioritize those patients who need urgent CA. In TC the ECG changes are new and usually localized in the precordial leads. The majority of patients with TC have an abnormal ECG.

ST-segment elevation is the most common ECG abnormality in 56% of the patients and T-wave inversion may be present in 39% of the patients [1]. Other types of ECG changes may develop, such as ventricular tachycardia, ventricular fibrillation, torsade de points, and QT interval prolongation that usually normalize within the first 48 h [1, 12]. According to Ogura et al., in patients with TC, ST-segment elevation in anterior leads (V1–V6) is higher than 1 mm, but without reciprocal ST-segment depression in inferior leads and without Q waves, that is very important for the differential diagnosis with AMI [13]. The same case series report showed that ST-segment elevation is higher in V4–V6 than in V1–V3 leads [13]. T wave inversion in precordial leads progresses gradually, having two negative peaks: a first negative peak at about three days and a second negative peak at approximately 2–3 weeks after disease onset [14]. The presence of prominent U waves is also considered to be in favor of the diagnosis of TC according to Vivo et al. [15]. Another study revealed that ST-segment depression in aVR associated with the lack of ST-segment elevation in V1 has good diagnostic accuracy (96% specificity, 91% sensitivity, 95% predictive accuracy) [16].

### Transthoracic echocardiography

TTE is often the first imaging investigation used in patients with TC suspicion and is very important for the diagnosis. The specific change in TC is the apical ballooning pattern of the LV appearing in the majority of the patients with TC (81.7%), because of the apical akinesia/dyskinesia and basal hyperkinesia [8]. The less frequent subtypes are present in the rest of patients: the mid-ventricular ballooning pattern (14.6%) because of the akinesia of the mid-LV segments, with normal kinesis of the apical and basal segments; basal pattern with motion abnormalities in the basal LV segments (2.2%); focal pattern with motion abnormality localized in a small area of the LV (1.5%) [8].

In contrast to AMI, in TC the RWMA is not limited to one epicardial coronary artery territory, which is a very useful evidence to support the diagnosis of TC.

In the acute phase of TC, LV systolic dysfunction is remarked, but compared to AMI, this dysfunction is transient with a complete recovery of the LV function [17]. Serial TTE is necessary to follow up the evolution of the LV function. According to Prasad et al., LV function improvement is seen at about 8 days from the onset [17].

TTE is also necessary to detect possible complications. In patients with apical ballooning subtype, systolic anterior motion and LV outflow tract obstruction (LVOTO) may be associated secondary to basal LV hyperkinesia. Mitral regurgitation may be secondary to papillary muscle dysfunction and leaflet tethering. Another possible complication is LV thrombus, which is most commonly located in the apical region. LV wall rupture is a rare, but extremely severe complication, with a high mortality rate.

### Cardiac computed tomography angiography

Cardiac computed tomography angiography is a non-invasive imaging modality, used in patients in whom invasive CA can not be performed. For example, septic shock or intracranial bleeding may trigger TC and in these situations, computed tomography angiography is preferred over CA [18]. It may also be used in old patients with important frailty or terminal malignancies in whom CA may have severe complications. Computed tomography may also be a better option in patients with a low probability of acute coronary syndrome, with known coronary anatomy due to previous CA, or in suspected recurrent TC in patients with previous CA [18]. It may be used in the emergency room for a rapid differential diagnosis between TC and AMI.

### Coronary angiography

CA is usually performed in most patients with TC to differentiate from AMI, especially in those with ST-segment elevation. Patients with TC have normal epicardial coronary arteries or have non-obstructive atherosclerotic stenoses (< 50%). CAD should always be excluded in order to establish the diagnosis of TC. Coexisting CAD may be present in about 15% of patients with TC, this is why the lesions detected by CA should be carefully correlated with echocardiographic RWMA [2]. Furthermore, a comparison between CA and biplane ventriculography in similar views is necessary to identify a possible perfusion-contraction mismatch to make the differential diagnosis between AMI and TC [18]. Left ventriculography shows the specific ballooning of the LV, confirming the diagnosis of TC. The “apical nipple sign” meaning a small segment with preserved contractility in the most distal part of the LV apex may appear in about one-third of the patients with TC [18].

Intravascular imaging tests, such as intravascular ultrasound or optical coherence tomography, can be used for a more accurate diagnosis. Myocardial infarction with non-obstructive coronary arteries due to coronary embolus, coronary dissection, or spasm is sometimes difficult to differentiate from TC, but a careful correlation between ECG, TTE, CMR, and CA would lead to a correct diagnosis. Left ventriculography reveals LV mid- and apical segments akinesis/dyskinesia associated with basal segments hypercontractility [19].

Even more, LV invasive hemodynamic measurements can be used to determine LV end-diastolic pressure that is increased. LVOTO is present in about 20% of the patients with TC [2]. LVOTO assessment by TTE or LV invasive hemodynamics is important for therapeutical management [20]. LV end-diastolic pressure has a prognostic impact and is a good predictor of complications during hospitalization [21].

### Cardiac magnetic resonance imaging (CMR)

CMR has a great utility in clinical practice to confirm the diagnosis of TC and to differentiate it from other similar cardiac pathologies, such as myocardial infarction with non-obstructive coronary arteries (MINOCA) and myocarditis, that may have similar ECG, echocardiographic and angiographic features as TC. CMR is usually recommended in patients with atypical clinical characteristics, non-obstructive CAD, and myocarditis suspicion. CMR is more accurate than TTE in assessing myocardial tissue structure and RWMA extended in multiple coronary arteries territories [8, 12]. Studies based on CMR revealed that TC may also affect the right ventricle in some patients, although it was initially thought that only LV is involved [22–24]. The CMR sequences used in patients with TC are early gadolinium enhancement, late gadolinium enhancement (LGE), and T2-weighted.

In patients with TC suspicion, CMR may be an imaging modality to confirm the diagnosis, especially in the acute phase [25, 26]. It may also detect some complications impossible to identify through other imaging investigations, for example, LV thrombi, that may not be seen by TTE. LV thrombi are identified using early gadolinium enhancement sequences, appearing as areas of low-intensity signal without gadolinium uptake, compared to the high-intensity signal of the blood [24, 27].

In the acute phase of TC, the typical CMR findings are the reversible acute myocardial inflammation and important edema, assessed by T2-weighted sequences [8, 23, 24, 28, 29]. Myocardial edema is transmural and multiple studies demonstrated that it resolves in approximately 6 months [22, 24, 27].

In the subacute phase, CMR may identify the fine remaining RWMA as a sign of the resolution of the initial regional severe LV dysfunction and later reveal the full LV function recovery [8].

LGE is usually absent in patients with TC and predicts the complete recovery of LVEF in these patients, but small areas of fibrosis and gadolinium fixation may be identified at the hinge points between the akinetic/dyskinetic ballooning segments and the hypercontractile adjacent segments in some cases [25, 27, 30, 31]. The absence of LGE in the dysfunctional LV segments allows the differential diagnosis between TC and MINOCA (subendocardial or transmural LGE corresponding to a specific coronary territory) or myocarditis (epicardial or patchy LGE) [25, 32–34].

### InterTAK diagnostic score

The Takotsubo International Registry proposed a clinical diagnostic score in order to assess the clinical probability of TC and try to differentiate it from AMI before imaging tests and CA are performed. According to Ghadri et al., the InterTAK Diagnostic Score has good specificity and sensitivity in TC diagnosis [35]. The InterTAK Diagnostic Score is shown in Table 3.

**Table 3:** InterTAK Diagnostic Score

InterTAK Diagnostic Score	
Female sex	25 points
Emotional stress	24 points
Physical stress	13 points
No ST-segment depression	12 points
Psychiatric illness	11 points
Neurological disorders	9 points
QT prolongation	6 points

## Management of patients with TC

There are no established guidelines for the treatment of patients with TC till now. Firstly, the management of patients with TC suspicion is similar to the management of those with AMI. After AMI is excluded and, taking into account that TC is a reversible pathology, supportive therapy of vital functions and careful monitoring in the intensive care unit in the acute phase is enough in most cases.

The treatment of TC includes beta-blockers, angiotensin-converting enzyme inhibitors (ACEI) or angiotensin II receptor blockers (ARBs), and mineralocorticoid inhibitors (if LV ejection fraction ≤ 40%). Beta-blockers have the additional effect of antagonizing the excess of catecholamines and preventing LV-free wall rupture but should be cautiously used in patients with QTc > 500 ms [8, 36]. ACEI has a cardioprotective effect due to renin angiotensin aldosterone system inhibition, central sympathetic system blocking, and bradykinin increase leading to vasodilation [37]. Statins may have a positive effect due to their anti-inflammatory effects, endothelial function improvement, and oxidative stress decrease and consequently have a favorable effect on mortality in patients with TC [37]. Antiplatelet therapy has no proven utility in the absence of CAD.

The study conducted by Petursson et al. using data from the SWEDEHEART Registry reviewed the effects of different drugs on mortality [37]. Therefore, in patients with TC, some drugs, such as inotropes, digoxin, and diuretics, were associated with increased mortality, while ACEI, statins, and anticoagulants (unfractionated heparin, low molecular weight heparin) lead to mortality decrease [37]. Other drugs—antiplatelets, beta-blockers, and ARBs did not influence the mortality [37]. Inotropes, such as dobutamine or dopamine, may worsen heart failure in patients with TC because catecholamines play an important role in TC pathophysiology and are associated with the highest 30-day mortality of all drugs [37]. In patients with TC who develop acute heart failure/pulmonary edema, intravenous diuretics and nitrates should be recommended, in the absence of LVOTO [2]. In patients with TC requiring inotrope support, levosimendan seems to be the optimal option because it has a different mechanism of action, and is a calcium sensitizer. Diuretics may increase mortality by renal injury or electrolyte disturbances and should be cautiously administered [37].

Cardiogenic shock is reported in more than 10% of patients with TC, leading to increased mortality [38]. LVOTO must be assessed by TTE or CA before initiating the therapy for cardiogenic shock. The use of exogenous catecholamines in patients with TC is limited only as a short-term bridge to LV circulatory mechanical support because they already have a high sympathetic tonus and catecholamines may worsen the prognosis and increase the mortality. Patients with TC and cardiogenic shock without LVOTO may receive the specific therapy with inotropes, preferably levosimendan, but LV mechanical support, such as intra-aortic balloon counterpulsation, temporary LV assist devices, or extracorporeal membrane oxygenation, is preferred [2].

In patients with TC and significant LVOTO, inotropes, diuretics, digoxin, and intra-aortic balloon pumps are contraindicated, because may worsen the obstruction[38]. Therefore, in these patients, intravenous fluids and short-acting intravenous beta-blockers are recommended [2, 38].

### LV thrombus

LV thrombus may appear in 1–2% of the patients with TC and require anticoagulation treatment to prevent embolization and thrombus increase for at least 3 months or until the thrombus is resolved [6, 8].

### Arrhythmias

General principles of acute management of ventricular arrhythmias are applicable in patients with TC. Antiarrhythmic drugs that prolong the QT segment should be avoided because may predispose to arrhythmias taking into account that patients with TC may present QT prolongation per se. High-degree atrioventricular block may appear in rare cases and temporary right ventricular pacing should be used until TC resolution [2].

Permanent pacing and inotropes should be avoided [2]. Cardiac defibrillators should be taken into account as a secondary prevention in patients with life-threatening ventricular arrhythmias [2].

### Long-term management

Complete recovery of LV function is a mandatory part of the diagnosis of TC and can occur in a few days or may last some weeks. Heart failure therapy should be maintained for 3 months or until LV function is completely recovered. ACEI has been shown to improve 1-year survival and decrease TC recurrence [2].

The differences regarding the treatment between patients with TC versus those with AMI are described in Table 4.

**Table 4:** Management differences between TC and AMI

	TC	AMI
9. Evolution and management		
Evolution		
Symptoms relieve	Complete after LV function recovery	Depends on LV function, ongoing ischemia, multivessel CAD, complete/incomplete revascularization
ECG	Normalize	AMI sequelae (Q waves), persistent T wave inversion, and persistent ST-segment elevation may appear
Echocardiography	LV function complete recovery, wall motion abnormalities remission	LV function complete/incomplete recovery, persistent/worse LV dysfunction
Treatment		
Acute phase complications		
Acute heart failure/pulmonary edema	Intravenous diuretics, nitrates (if LVOTO is absent)	Intravenous diuretics, nitrates
Cardiogenic shock	LV assist device, venous to arterial extracorporeal membrane oxygenation AVOID: inotropes- epinephrine, norepinephrine, dobutamine, milrinone, isoprenaline	-LV assist device, venous to arterial extracorporeal membrane oxygenation -Inotropes-epinephrine, norepinephrine, dobutamine, milrinone, isoprenaline on short term
LVOTO	Beta-blockers, intravenous fluids in the absence of acute heart failure AVOID: diuretics, nitrates, intra-aortic balloon pump	–
Arrhythmias	Ventricular arrhythmias: beta blockers, magnesium sulfate, electrical cardioversion; AVOID QT-prolonging drugs High degree atrioventricular block: temporary pacing; AVOID: permanent pacing, beta-blockers	Ventricular arrhythmias: beta blockers, magnesium sulfate, amiodarone, lidocaine, electrical cardioversion High degree atrioventricular block: temporary pacing
LV thrombus	Anticoagulation for at least 3 months	Anticoagulation for at least 3 months
In-hospital/at discharge		
Antiplatelets	−	+
Statins	+	+
Beta-blockers	+	+
ACEI	+	+
ARBs	±	+
Mineralocorticoid inhibitors	± (if LVEF< 40%)	± (if LVEF< 40%)
Diuretics	± (if heart failure symptoms)	± (if heart failure symptoms)
Digoxin	−	−
Nitrates	−	± (antianginal effect)
Antiarrhythmic drugs	Beta-blockers, ivabradine AVOID: QT prolongation drugs	Beta-blockers, amiodarone
1.Prognosis		
	Usually benign, but severe complications may occur	Reserved

*ACEI* Angiotensin-converting enzyme inhibitors, *AMI* Acute myocardial infarction, *ARBs* Angiotensin receptor blockers, *CAD* Coronary artery disease, *LV* Left ventricle, *LVOTO* Left ventricle outflow tract obstruction, *LVEF* Left ventricle ejection fraction, *TC* Takotsubo cardiomyopathy

### Association between TC and AMI

There are several reported cases of patients with TC and AMI [39–41]. Commonly in these cases, is considered that the AMI is the trigger for developing TC due to acute stress, sympathetic system activation, and catecholamine release [39–41]. Post-ischemic myocardial stunning or extreme pain related to AMI are other possible factors for TC development. The incidence of TC-like dysfunction of the LV caused by left anterior descendent artery occlusion is about 26% according to some studies and is more common in women [39].

### Post-operative TC

Postoperative acute stress following surgical intervention/procedures may precipitate the development of TC due to catecholamine discharge. Initially, the ECG and echocardiographic characteristics may mimic perioperative AMI with LV systolic dysfunction. It may occur in any type of invasive or minimally invasive procedure. Stress response to surgery begins during inducing general anesthesia and may last till 3 or 4 days after the surgery, predisposing to TC development [42]. TC should be taken into account especially in post-menopausal female patients with a suspicion of AMI as a differential diagnosis. The methods that may reduce TC incidence in these patients are pain and anxiety management, permanent monitoring of the ECG and cardiac function (through troponin monitoring in dynamics and comparing with preoperative values, intra-operative transesophageal echocardiography if necessary or postoperative serial TTE), maintaining a normovolemic status and normal blood gases [42]. In rare cases, TC may develop after cardiac surgery.

The systematic review by Laghlam et al. analyzed the incidence and the evolution of patients with TC after cardiac surgery [43]. The review reported a lower postoperative incidence of TC of only 0.1% and the majority of the patients were women (79% of the patients) [43]. Usually, TC develops early after surgery, but in rare cases, it may appear later. Post-operative TC was related mostly to surgery procedures implying atrioventricular valves, but it may occur in any type of cardiac surgery [43]. It was noticed a 6% in-hospital mortality, a mean period of intensive care unit hospitalization after surgery of 5–12 days in patients who developed TC [43]. Complete recovery, with an LV ejection fraction of 50–60% on discharge was observed in most of the patients [43].

## Prognosis

TC is characterized by a complete recovery of the LV function and in most cases is considered to be a benign disease, with a good short- and long-term prognosis. However, patients with TC should be carefully followed up during hospitalization by ECG and echocardiography and afterward until LV function complete recovery, because they have a greater potential of recovery than those with AMI.

In the acute phase, the possible complications of TC are comparable to complications that may appear in patients with AMI, such as acute heart failure, cardiogenic shock, life-threatening arrhythmias, and cardiac arrest [44]. The rate of recurrence between 3 weeks and 3.8 years is about 5% [44].

Patients with TC triggered by physical effort, medical conditions, or procedures have a worse prognosis, with a 3 times higher long-term mortality rate compared with patients with TC triggered by emotional stress [2]. The higher long-term mortality is observed in patients with TC induced by neurological diseases [2]. In-hospital mortality in patients with TC is about 4.5%, which is comparable with mortality in patients with ST-segment elevation myocardial infarction [43].

## Conclusions

Initially, the therapeutical approach in patients with TC should be similar to in patients with AMI, with vital functions support and monitoring in the intensive care unit. AMI is the major emergency that requires prompt myocardial revascularization, thus the patients with diagnostic suspicion of TC versus AMI are initially treated as an AMI for their benefit. The differential diagnosis between TC and AMI is immediately necessary. Clinical presentation may be similar between TC and AMI, but paraclinical tests reveal differences, such as ST-segment elevation in precordial leads without reciprocal ST-segment depression in inferior leads on the ECG, RWMA that are not localized in one epicardial coronary artery territory on TTE. In some cases, ECG and TTE features of TC may mimic AMI, therefore CA is usually performed in order to exclude obstructive CAD. Coronary computed tomography angiography may be taken into account as an alternative method of diagnosis in special situations, such as in patients with a low probability of AMI or in those with septic shock or intracranial bleeding. After the clinical and paraclinical data indicate the diagnosis of TC, the management is different from AMI.

Although TC is considered a benign condition, severe complications, such as cardiogenic shock, life-threatening arrhythmias, LVOTO, and cardiac arrest, may develop and must be treated urgently. The management of these complications is different for patients with AMI, for example, in cardiogenic shock, it is preferable to use mechanical circulatory support from the beginning, in ventricular arrhythmias QT prolonging drugs should be avoided.

In patients with LVOTO, intravenous fluids and beta-blockers should be considered and diuretics, nitrates, inotropes, and intra-aortic balloon pumps should be avoided. An optimal therapeutical approach and careful monitoring are very important in patients with TC because they have a higher LV function recovery potential compared to patients with AMI.

## Data Availability

No datasets were generated or analysed during the current study.

## Abbreviations

AMI: Acute myocardial infarction
BNP: Brain natriuretic peptide
CA: Coronary angiography
CAD: Coronary artery disease
ECG: Electrocardiogram
HT: Arterial hypertension
LV: Left ventricle
LVOTO: Left ventricle outflow tract obstruction
MINOCA: Myocardial infarction with non-obstructive coronary arteries
NT-proBNP: N-terminal proBNP
SAM: Systolic anterior motion
TC: Takotsubo cardiomyopathy
TTE: Transthoracic echocardiography
